# Decreases in Community Viral Load Are Accompanied by Reductions in New HIV Infections in San Francisco

**DOI:** 10.1371/journal.pone.0011068

**Published:** 2010-06-10

**Authors:** Moupali Das, Priscilla Lee Chu, Glenn-Milo Santos, Susan Scheer, Eric Vittinghoff, Willi McFarland, Grant N. Colfax

**Affiliations:** 1 San Francisco Department of Public Health, San Francisco, California, United States of America; 2 University of California San Francisco, San Francisco, California, United States of America; Institute of Human Virology, United States of America

## Abstract

**Background:**

At the individual level, higher HIV viral load predicts sexual transmission risk. We evaluated San Francisco's community viral load (CVL) as a population level marker of HIV transmission risk. We hypothesized that the decrease in CVL in San Francisco from 2004–2008, corresponding with increased rates of HIV testing, antiretroviral therapy (ART) coverage and effectiveness, and population-level virologic suppression, would be associated with a reduction in new HIV infections.

**Methodology/Principal Findings:**

We used San Francisco's HIV/AIDS surveillance system to examine the trends in CVL. Mean CVL was calculated as the mean of the most recent viral load of all reported HIV-positive individuals in a particular community. Total CVL was defined as the sum of the most recent viral loads of all HIV-positive individuals in a particular community. We used Poisson models with robust standard errors to assess the relationships between the mean and total CVL and the primary outcome: annual numbers of newly diagnosed HIV cases. Both mean and total CVL decreased from 2004–2008 and were accompanied by decreases in new HIV diagnoses from 798 (2004) to 434 (2008). The mean (p = 0.003) and total CVL (p = 0.002) were significantly associated with new HIV cases from 2004–2008.

**Conclusions/Significance:**

Reductions in CVL are associated with decreased HIV infections. Results suggest that wide-scale ART could reduce HIV transmission at the population level. Because CVL is temporally upstream of new HIV infections, jurisdictions should consider adding CVL to routine HIV surveillance to track the epidemic, allocate resources, and to evaluate the effectiveness of HIV prevention and treatment efforts.

## Introduction

In individuals, antiretroviral therapy (ART)-mediated virologic suppression reduces perinatal transmission [1]–[5] and may reduce sexual transmission [6]–[12]. However, it remains unclear whether increasing HIV testing and the levels of ART uptake can reduce HIV incidence on a population- level. In some areas of the world, the widespread introduction of ART that occurred in the late 1990s was accompanied by decreases in new HIV infections as measured by new HIV diagnoses in surveillance data or HIV incidence in contemporaneous cohort studies. In Taiwan, for example, the expansion of ART was associated with a greater than 50% reduction in reported new HIV cases [13]. In Vancouver, a temporal trend in reduction of median VL among HIV-positive injection drug users (IDU) in care was observed in parallel to a reduction in HIV incidence in a cohort study of HIV-negative IDU [14]. However, in other jurisdictions, risk compensation or increases in sexual risk behavior with the introduction of ART may have blunted the preventive effect, a possible explanation for a rise in HIV incidence among men who have sex with men (MSM) in San Francisco and elsewhere [15], [16]. Indeed, different modeling approaches using varied assumptions regarding adherence, percent coverage of ART-eligible individuals, survival, duration of infectiousness, and risk compensation arrive at different conclusions regarding the net effect of ART on preventing HIV transmission at the population level [17]–[22]. In this paper, we evaluate “community viral load” (CVL) as a biological marker of ART-mediated virologic suppression and HIV transmission potential at the population level. The latter implies the hypothesis that suppressing CVL would reduce population HIV incidence.

The current new era of highly potent, tolerable ART,[23] coupled with recent advances in HIV testing technology and policy changes such as recommendations for routine testing, have increased the proportion of HIV-infected individuals who are aware of their diagnosis[24]–[26] and receiving ART [27]–[29]. In San Francisco, the implementation of public health measures[30] including increasing identification of acute infections[31], [32], simplifying HIV testing procedures [26], [33], increasing partner notification[34], [35] and linkage to care[36] has increased both testing and ART uptake. Both testing frequency and the proportion of HIV-infected individuals aware of their serostatus increased from 2004 to 2008 according to data from the National HIV Behavioral Surveillance (NHBS) surveys [37]. In 2008, 72% of MSM (the predominant risk group in San Francisco) received an HIV test in the past 12 months compared to 65% in 2004, with over half (53%) testing in the past 6 months in 2008 (local unpublished data from one of the co-authors, McFarland). The proportion of HIV-positive MSM unaware of their HIV status decreased from 23% in 2004 to between 20.0% and 15.1% in 2008 (local unpublished data from McFarland). Most (88%) newly diagnosed individuals were linked to primary care within 3 months of diagnosis [36]. A greater percentage of HIV-positive persons were in care in 2008 (78%) as compared to 2004 (71%) [27]–[29]. Notably, the proportion of people living with AIDS who were receiving ART increased from 74% in 2005 to 90% in 2008 [29]. However, measures of population-level sexual risk behavior, such as cases of rectal gonorrhea among MSM, an indicator of unprotected receptive anal intercourse, increased from 400 in 2004 to 465 in 2008 [29].

Our primary hypothesis is that in the current era of more effective ART, increased uptake of HIV testing, and improved linkage to care, a greater number of individuals will be virologically suppressed, which in turn will lower CVL, and ultimately reduce new HIV infections. Building on the findings of Wood *et al.* that a reduction in median VL among HIV-positive IDU in care correlated with reduced HIV incidence in a cohort study of HIV-negative IDU [14], we extended the concept using San Francisco's comprehensive, population-based HIV/AIDS case surveillance system. Using surveillance data, which includes mandatory laboratory-based reporting of all VL results from patients diagnosed with HIV, we calculated two measures of CVL, the mean and total of the most recent VL results. We compared these CVL measures across populations affected by HIV, examined their spatial distribution, and assessed the correlation between CVL and newly diagnosed and reported HIV cases from 2004 to 2008. We also examined temporal correlations between CVL and population rates of virologic suppression, as well as estimated HIV incidence.

## Methods

### Ethics Statement

The Department of Health and Human Services (DHHS) regulations for the protection of human subjects in 45 CFR 46.101(b)(4) qualifies this research as exempt from IRB review because it uses existing, de-identified data. Furthermore, the coded private information was not collected for the current research project, and the primary investigator and the holder of the data key entered into an agreement prohibiting the release of the key under any circumstances. Therefore, according to the University of California, San Francisco's Committee on Human Research, this research is not considered *human subjects* research and does not require IRB review or exempt certification.

HIV is a mandatory reportable disease in California. The data collected for the HIV/AIDS Surveillance registry were part of routine core public health disease surveillance which includes the legally mandated reporting of all viral loads. The datasets used for this study were de-identified prior to analyses, the data were coded and identifiers were kept separately, and the persons involved in the analyses did not have access to the “key” to link back to the names contained in the HIV/AIDS surveillance registry.

### Measures and definitions

#### San Francisco HIV/AIDS Case Surveillance System VL Data

Systematic VL reporting by laboratories began with the implementation of mandatory names-based HIV case reporting in California in April 2006. Subsequently, the San Francisco registry of HIV/AIDS cases has been validated to include over 95% of all HIV/AIDS diagnoses in the county [38]. Using a combination of active (chart review) and passive (electronic and provider-initiated case reports) surveillance, we collected and examined treatment history, demographic characteristics, transmission risk, health insurance status, most recent CD4 count, history of ART regimen change, hepatitis C infection, engagement in care, and country of origin for all diagnosed HIV/AIDS cases in San Francisco. Sequential VL results were recorded prospectively and retrospectively from the initially reported diagnoses. The datasets used for the analyses reported here were de-identified.

#### Community Viral Load

We used the term CVL to refer to an aggregate biological measure of VL for a particular geographic location—for example the city of San Francisco or a particular neighborhood— and for a particular group of people who share socio-demographic characteristics. Although IDU, MSM, or specific ethnicities may not necessarily constitute a “community” in the sense of complete social interconnectedness or shared networks, we used the term to broadly refer to populations defined by demographic, geographic, or behavioral commonality with the potential for a higher probability of connections to similar members of the population, including those through needle-sharing or sexual partnerships. Individuals were included in the calculation of mean and total CVL for the subpopulation and geographic analyses if they had one or more VL between 2005 and 2008 regardless of current treatment status or engagement in care. This time period was chosen to ensure that we included VL data from the greatest number of people in the HIV/AIDS surveillance registry after the institution of both names-based HIV and electronic VL reporting.

#### Mean Community Viral Load

We defined mean CVL as the average of the most recent VL of all reported HIV-positive individuals in a particular population in each year from 2005 to 2008 for the purpose of exploring mean CVL among sub-populations and by neighborhood. We then calculated annual mean CVL from 2004–2008 for examining temporal trends and associations with virologic suppression, newly reported HIV infections, and HIV incidence estimates. For individuals with multiple, repeated VL measures, the most recent VL was used. Mean CVL is an indicator of the average viral burden for a particular population of HIV-positive persons, and should reflect treatment effectiveness and transmission risk under our primary hypothesis. Furthermore, the mean is a stable and approximately normal estimate of central tendency in samples as large as ours, despite the substantial right skewness of viral load values. Median CVL would also be a robust measure of central tendency, but could lack a direct relationship to population transmission rates. For example, in a hypothetical population in which the prevalence of viral suppression increases from 60% to 80% over 5 years, median viral load would be below the limit of detection every year and thus unchanged. In contrast, mean CVL would reflect the increases in suppression, and has a direct relationship to total community viral load, possibly a more important population marker of potential transmission. Specifically, in our dataset, greater than 50% of individuals had evidence of virologic suppression from 2004–2008 and thus the median CVL was < = 75 copies/mL annually from 2004–2008; the lack of variability in the median limited our ability to examine the relationship between median CVL and new HIV infections.

#### Total Community Viral Load

We defined total CVL as the sum of the most recent VL of all reported HIV-positive individuals in a particular population from 2005–2008 for the geographic analysis. We then calculated annual total CVL measures for evaluating the association with population rates of virologic suppression, new HIV diagnoses, and HIV incidence. The total CVL serves as a measure of the absolute level of virus in a population at the most recent point in time, and is therefore a gauge of potential infectiousness in a particular population. The measure takes into account both the number of individuals with HIV/AIDS in a particular population as well as each individual's most recent viral load.

#### New HIV diagnoses

We defined new HIV diagnoses by the year of the earliest known HIV-positive test result for all HIV/AIDS cases reported annually from 2004 to 2008. New HIV diagnoses became reportable by name in California in 2006; previous code-reported cases were re-reported and reclassified by name as far back as 2004. We recognize that new HIV diagnoses may sometimes represent newly diagnosed but chronically infected individuals, rather than incident HIV infections. Nonetheless, trends in new HIV diagnoses have been used as a marker for HIV incidence in previous studies in Taiwan [39] and Australia [40], [41], and have been consistent with trends in estimates of HIV incidence in San Francisco based on other methods [42].

#### HIV Incidence

Applying the CDC methods used to estimate national HIV incidence [43], San Francisco calculated the local HIV incidence estimates for 2006–2008 [42], which were used as outcome measures in the present analysis. The time period is restricted to start in 2006 because San Francisco was funded to conduct HIV incidence surveillance only after names-based HIV reporting was adopted in 2006. The method, previously described in detail [44], estimates HIV incidence based on the number of recent HIV infections detected by the Serologic Testing Algorithm for Recent HIV Seroconversion (STARHS) among newly reported cases in the surveillance system while adjusting for the likelihood of testing, use of ART, missing specimens, and the duration of the BED assay window period (within the previous 6 months). The BED and testing history are used to estimate prevalence among non-testers and impute data for testers to calculate a population-based incidence estimate. We have previously reported that the method produced local HIV incidence estimates comparable to other methods used in San Francisco [28], [29].

#### Percent Virologic Suppression ≤75 copies/mL

We define virologic suppression as having the most recent VL ≤ 75 copies/mL, which was the lower limit of detectability in the earlier part of the time period analyzed.

### Analysis

We conducted two sets of analyses: a cross-sectional period analysis using all the data from 2005–2008 and longitudinal analyses using annual data from 2004 to 2008. We used the cross-sectional data from the time period from 2005 to 2008, to calculate mean and total CVL for San Francisco as a whole as well as for sub-populations defined by demographic characteristics, transmission risk category, clinical factors, and geography. We used Geographic Information Systems software (ArcGIS v 9.3) to map the cross-sectional 2005–2008 mean and total CVL data by neighborhood. Differences in this initial cross-sectional estimate of mean CVL for various populations were assessed using the Kruskal-Wallis test. We then calculated the annual measures of mean and total CVL from 2004 to 2008 and used Poisson models to assess the relationships between these two annual CVL measures and newly reported HIV diagnoses in 2004–2008; robust standard errors, which relax the Poisson assumption that the variance of the outcome equals its mean, were used to account for potential over-dispersion. Linear models were used to assess the relationship of annual measures of mean and total CVL with population rates of virologic suppression from 2004–2008. We assessed the relationships between trends in mean and total CVL and estimated HIV incidence from 2006–2008 using meta-regression to account for the imprecision of the CDC HIV Incidence estimates. Analyses were repeated using log-transformed mean and total CVL, without materially affecting results.

While over 95% of HIV/AIDS diagnoses made in San Francisco are included in the surveillance database, many observations have missing data; in particular, VL was only 74.4% complete. We attempted to characterize the individuals who were missing VLs. Of those 4,312 individuals missing VLs, 4,262 (99%) were not in engaged in care in San Francisco; of whom 569 could be confirmed to have moved out of the jurisdiction. One large private medical provider that cares for 3,142 HIV-infected patients in the analysis dataset only reports the initial diagnosis VL to the HIV/AIDS surveillance registry; 691 individuals seen at this institution were missing VLs. Analyzing only complete cases may induce bias and wastes information available from observations with incomplete data. To avoid these problems, we used SAS Proc MI to multiply impute VL on the log scale based on race/ethnicity, sex, gender, age at diagnosis, transmission risk category, insurance status, and neighborhood, as well as CD4 count, treatment history, AIDS diagnosis, Hepatitis C co-infection, number of opportunistic infections, history of ART use, and engagement in care. The ten completed datasets were then analyzed using SAS Proc MIAnalyze, which combines the dataset-specific results using established methods that provide standard errors, confidence intervals, and p-values properly reflecting imputation of missing data [45]. As a sensitivity analysis, we conducted multiple imputation to generate VL results excluding the individuals who had moved out of the jurisdiction.

## Results

The overall San Francisco mean CVL was 23,348 copies/mL for the 12,512 unduplicated HIV-positive individuals reported in the surveillance database and who had at least one VL during the cross-sectional analysis time period 2005 through 2008. Table 1 shows mean CVL for different populations according to reported VL and with imputation for missing VL (total missing 4,312) for the time period 2005–2008. Neither the relative mean CVL nor variations in the mean changed when using the imputed values. Similarly, in the sensitivity analyses, excluding those who had moved did not change the mean CVL nor variations in the mean. Remaining findings are therefore presented using the observed data.

**Table 1:** Characteristics of HIV-positive persons and mean community viral load (CVL), San Francisco, 2005–2008.

Variable	Subcategory	N (%)	Mean CVL	Kruskal-Wallace	Mean CVL, imputed data
**Overall**		12,512	23,348		
**Treatment history**				<0.001	
	On treatment	9,588 (77)	18,252		17,200
	Not on treatment	2,924 (23)	40,056		44,149
**Race/ethnicity**				<0.001	
	White	8,019 (64)	21,087		22,596
	African American	1,825 (15)	26,404		30,192
	Other^1^	846 (7)	30,807		31,577
	Latino	1,822 (15)	26,774		27,490
**Age in years**				<0.001	
	0–19	96 (1)	33,621		34,542
	20–29	2,379 (19)	33,077		33,360
	30–39	5,436 (54)	23,348		24,236
	40–49	3,473 (21)	18,717		21,161
	50–59	951 (7)	15,725		18,997
	60+	177 (1)	18,824		26,663
**Sex**				<0.001	
	Male	11,726 (94)	23,062		24,651
	Female	786 (6)	27,614		31,234
**Transgender**				<0.001	
	Yes	291 (2)	64,160		58,951
	No	12,221 (98)	22,376		24,238
**Transmission risk**				<0.001	
	IDU	1,011 (8)	33,245		38,592
	MSM/IDU	1,791 (14)	36,261		36,764
	Other^2^	713 (6)	24,221		29,919
	MSM	8,997 (72)	19,596		20,799
**Insurance status**				<0.001	
	Public	1,568 (13)	26,286		28,661
	Private	6,155 (49)	16,577		18,088
	None	3,513 (28)	27,936		27,980
	Other/Unknown^3^	1,276 (10)	39,765		40,497
**Most recent CD4 count**				<0.001	
	<50	368 (3)	124,318		128,300
	50–199	1,391 (12)	27,052		46,706
	200–349	2,547 (21)	23,347		23,316
	> = 350	7,730 (64)	12,994		13,087
**Ever changed regimen**				<0.001	
	Yes	2,167 (17)	18,835		18,637
	No	10,345 (83)	24,293		26,015
**Hepatitis C**				<0.001	
	Yes	2,347 (19)	27,498		28,669
	No	10,165 (81)	22,389		24,368
**Engaged in care**				<0.001	
	Yes	7,875 (63)	15,314		15,309
	No	4,637 (37)	36,992		33,668
**Country of origin**				0.0452	
	US	10,307 (82)	23,812		25,033
	Non-US	1,395 (11)	23,428		24,433
	Other/Unknown	810 (6)	17,305		26,218

1 Other race includes Asian, Pacific Islander, Native American, Mixed race, Other, and Unknown.

2 Other risk includes sex with female, hemophilia, blood transfusion, heterosexual relations, transplant, occupational, and no identified risk.

3 Other/Unknown insurance includes VA, Healthy San Francisco, San Francisco Jail, and unknown insurance status.

There were statistically significant variations (p<0.001 by Kruskal-Wallis test) in mean CVL by treatment history, race/ethnicity, age, gender, HIV transmission risk category, insurance status, and clinical status. Mean CVL for African-Americans (26,404 copies/mL; N = 1,825; 15%), for Latinos (26,774; N = 1,822; 15%), and for women (27,614 copies/mL; N = 786; 6%) were higher than the overall San Francisco mean CVL. Transgendered individuals had also had a higher mean CVL (64,160 copies/mL; N = 291, 2%) than the city as a whole. IDU (33,245 copies/mL; N = 1,011; 8%) and MSM-IDU (36,261 copies/mL; N = 1,791; 14%) had higher mean CVL than the overall mean, whereas MSM (19,596 copies/mL; N = 8,997; 72%) had the lowest. Persons on treatment (18,252 copies/mL; N = 9,588; 77%) and those who were engaged in care (15,314 copies/mL; N = 7,875; 63%) had a lower mean CVL than the overall mean (23,348 copies/mL) and those not currently on treatment (40,056 copies/mL; N = 2,924; 23%) or not engaged in care (36,992 copies/mL; N = 4,637; 37%). Mean CVL was highest among the groups of people with the lowest CD4 counts, linearly decreasing as CD4 count increased.

**Figure 1:**
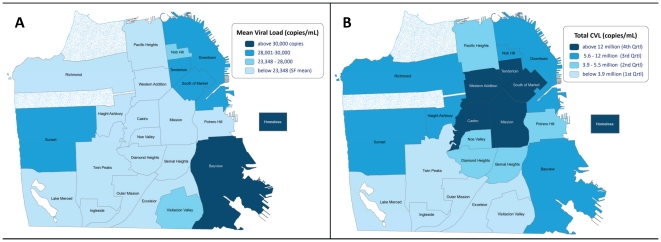
Spatial Distribution of CVL by Neighborhood, 2005–2008. Neighborhood mean (1a) and total CVL (1b) are shown. Mean CVL was highest among homeless individuals (38,974copies/mL; N = 775; 6%). The highest mean CVL (38,428 copies/mL; N = 278; 2%) was in the southeast neighborhood of Bayview, which is characterized by lower income and a predominantly African-American population. The northeast and inner city areas of the Tenderloin and South of Market (characterized by low income and large numbers of IDU, commercial sex workers, and transgendered persons) also had a mean CVL above the municipal average (28,093 copies/mL; N = 1,486; 12%). The Castro neighborhood (an historic gay and relatively upper-income neighborhood with very high HIV/AIDS case density) had a mean CVL of 21,352 copies/mL (N = 2,106; 17%), below the city as a whole. The distinction between mean CVL and total CVL is seen in 1b. The highest total CVLs are evident in the Tenderloin, South of Market, Mission, and the Castro, where there are either large numbers of persons living with HIV, many persons with high VLs, or a combination thereof.

Total CVL reflected mean CVL by population with a notable exception of geography. The highest mean CVL (38,428 copies/mL; N = 278; 2%) was in the southeast neighborhood of Bayview, which is characterized by lower income and a predominantly African-American population (Figure 1a). Homeless persons had the highest mean viral load (38,974 copies/mL; N = 775; 6%). The northeast and inner city areas of the Tenderloin and South of Market (characterized by low income and large numbers of IDU, commercial sex workers, and transgendered persons) also had a mean CVL above the municipal average (28,093 copies/mL; N = 1,486; 12%). Of note, the Castro neighborhood (an historic gay and relatively upper-income neighborhood with very high HIV/AIDS case density) had a mean CVL of 21,352 copies/mL (N = 2,106; 17%), below the city as a whole. The distinction between mean CVL and total CVL is seen in Figure 1b. The highest total CVLs are evident in the Tenderloin, South of Market, Mission, and the Castro, where there are either large numbers of persons living with HIV, many persons with high VLs, or a combination thereof.

**Figure 2:**
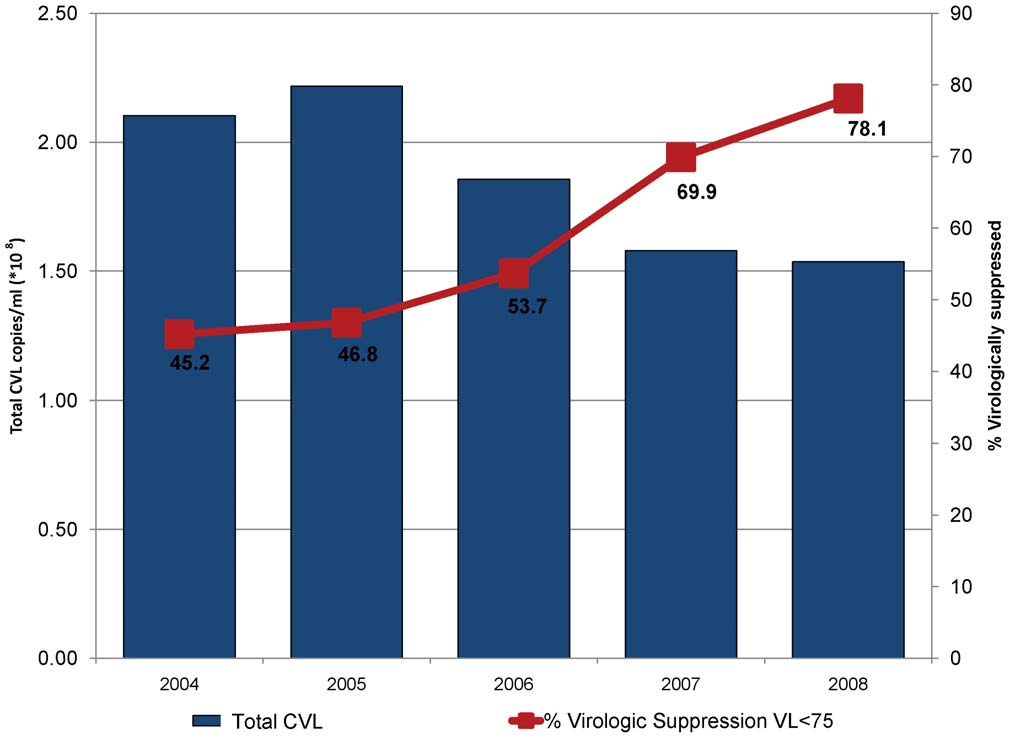
Total CVL and Virologic Suppression, 2004–2008. There was a statistically significant decline in annual measures of total CVL from 2004–2008 (p = 0.021). As expected, there was an inverse correlation between the increase in virologic suppression (red line) from 45% in 2004 to 78% in 2008 (p = 0.006) and total CVL (p = 0.011).

**Figure 3:**
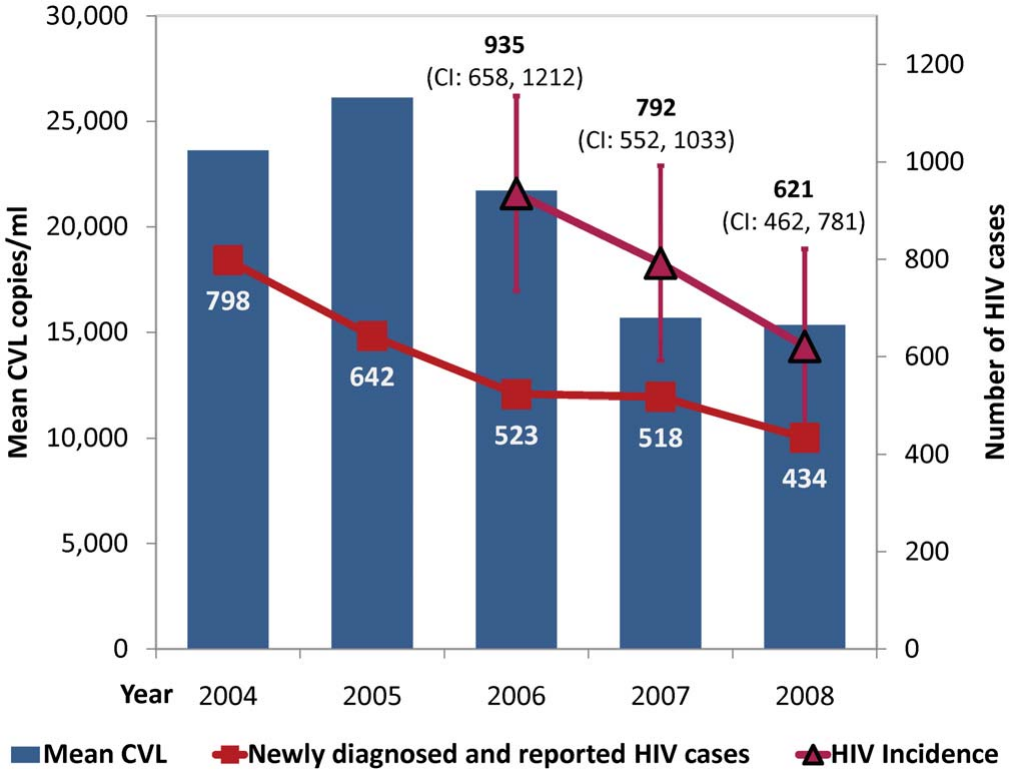
Mean CVL and New HIV Infections, 2004–2008. There was a statistically significant decline in annual measures of mean CVL from 2004–2008 (p = 0.037). Newly diagnosed cases of HIV (shown in red with 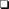) decreased in San Francisco from 798 (2004) to 434 (2008) (p<0.005). The point estimates of HIV incidence (shown in dark red with ▵) using the CDC methods also declined from 935 [95% CI 658–1212] in 2006, to 792 [552–1033] in 2007 and 621 [462–781] in 2008, although the change was not statistically significant (trend p = 0.29). The reductions in annual measures of mean CVL were significantly associated with decreases in newly diagnosed and reported HIV cases from 2004–2008 (p = 0.003). Longitudinal reductions in estimated HIV incidence were consistent with the trends in mean and total CVL, but the association in the meta-regression was not statistically significant (p>0.3).

From 2004 to 2008, there were statistically significant declines in annual measures of both total (p = 0.021) and mean CVL (p = 0.037) as shown in Figure 2 and Figure 3. As expected, there was an inverse correlation between the increase in virologic suppression from 45% in 2004 to 78% in 2008 (p = 0.006) and both total CVL (p-0.011, shown in Figure 2) and mean (p = 0.013, not shown). Newly diagnosed cases of HIV decreased in San Francisco from 798 (2004) to 434 (2008) (p<0.005). The point estimates of HIV incidence using the CDC methods also declined from 935 [95% CI 658–1212] in 2006, to 792 [552–1033] in 2007 and 621[462–781] in 2008, although the change was not statistically significant (trend p = 0.29, Figure 3). The reductions in annual measures of mean CVL were significantly associated with decreases in newly diagnosed and reported HIV cases from 2004–2008 (p = 0.003, shown in Figure 3) as were reductions in annual measures of total CVL (p = 0.002, not shown). Longitudinal reductions in estimated HIV incidence were consistent with the trends in mean and total CVL, but the association in the meta-regression was not statistically significant (p>0.3).

## Discussion

We found that the decreases in annual measures of mean and total CVL in San Francisco were significantly associated with temporal decreases in the number of new HIV diagnoses and accompanied by a decline in estimated HIV incidence by over one-third from 2006–2008. Although the trend in reduction of HIV incidence was not statistically significant, due to several sources of variation in the estimation, the trend across three consecutive years is consistent with the parallel and highly statistically significant decline in newly reported and diagnosed HIV cases and with trends in several indicators of potential transmission. A particularly rapid decrease in CVL occurred from 2005 to 2008, a period characterized by an increase in ART uptake among people living with AIDS from 74%[28] to 90% [29], availability of more effective, potent, and tolerable ART regimens, and a significant increase in the population rate of virologic suppression from 46.8% of all HIV/AIDS patients in 2005 to 78.1% by 2008 (Fig. 2). The trends are also concurrent with changes in laws facilitating HIV testing [26],policy initiatives promoting expanded HIV testing [25], [26], increasing acute HIV detection [46], [47], partner services [34], [35], and a reduction in the rate of unknown HIV infection. Taken together, the findings support our primary hypothesis that reductions in CVL resulting from increased ART uptake, expanded ART options, and greater virologic suppression can in turn reduce HIV incidence at the population level.

Our analysis also supports the use of CVL as a surveillance biomarker for treatment effectiveness and HIV transmission risk in a given jurisdiction or among a group of individuals. Differences in mean CVL among different groups are consistent with our understanding of the known disparities in HIV/AIDS morbidity and mortality in San Francisco, particularly with respect to low-income neighborhoods, African-Americans, and transgendered individuals [29], [48], [49]. The geographic disparities in CVL are consistent with known disparities in socioeconomic status and may also reflect neighborhood-level environmental factors [48], [50], [51] that structure HIV risk and access to prevention and care, such as availability of alcohol and drugs, transportation, and proximity to quality health care programs. These factors may be most evident in the low-income and relatively isolated Bayview with its very high mean CVL. As expected, the Castro had the overall highest total CVL given its high HIV/AIDS case density. Yet, the high total CVL in the Tenderloin is worthy of further exploration as it likely reflects both a high number of persons living with HIV coupled with disparities in any of several factors, including the uptake of ART, adherence to ART, or engagement in care. Our findings suggest that targeting HIV prevention to areas with the greatest total CVL, such as the Castro, should produce the greatest reductions in overall HIV incidence, while addressing inequities in both access to care and prevention services should decrease the relative disparities in HIV incidence among the subpopulations and in the geographic areas in San Francisco with highest mean VL.

There are several limitations to our study, including the well-understood problems with surveillance data. In particular, our dataset only included HIV-positive individuals who were diagnosed and reported in the HIV/AIDS case registry and had at least one VL measurement between 2005 and 2008. We were not able to include individuals who are not yet diagnosed. However, the estimated 15–20% of HIV-positive persons who are unaware of their infection is lower in San Francisco than the 25% unaware nationally in 2004 [24]. Our analysis also does not include those who are diagnosed but not yet reported to the HIV/AIDS case registry, but this is estimated to be less than 5% of all cases [38]. We also could not include all persons who are acutely infected with HIV, who may contribute significantly to amplified HIV transmission [52], [53], as many of these individuals are not diagnosed in the acute stage of infection. Omission of acute infections would affect the longitudinal trend in mean or total CVL if (1) acute infections compose a large proportion of the total number of cases or (2) the rate of new infections rapidly changed. However, we currently estimate that acute infections comprise a small minority of all prevalent HIV infections [46], [47] in San Francisco. If we were able to improve the detection of acute HIV infections and they are as important a part of transmission as hypothesized by some investigators [52], [53], we might even see a stronger correlation between CVL and incidence rates. The increase in the correlation would be very slight, however, because acutely infected persons are a very small minority of the total population included in CVL. In addition, viral load results were missing for 26% of persons in the registry. We used multiple imputation to replace those missing values, but this requires the strong assumption that the data are missing at random, given the available covariates. This assumption may not hold, so that the MI estimate of mean CVL in any given year remains biased. However, the associations between trends in CVL and new HIV infections could be approximately unbiased, provided the bias is comparable across years. Another limitation is that HIV incidence estimates calculated by the CDC method involve several imprecisely estimated inputs. For example, not all recent HIV diagnoses have remnant samples available for BED assay testing, resulting in an imprecise assessment of recent vs. chronic infection; similarly, not all cases have HIV testing history reported, which is needed to adjust incidence estimates. Thus, while point estimates of HIV incidence declined by over one-third, neither the trend nor the association with CVL reached statistical significance. In addition, we did not have the power to examine whether differences in mean CVL by sub-group were related to disparities in HIV incidence by sub-group. We also acknowledge the important possibility of ecologic fallacy. This is an observational study and there is limited ability to directly assess causality. For example, we cannot determine if transmissions occur from treated or untreated individuals. Similarly, the association between community viral load and new HIV infections could be confounded by different levels of risk behavior among distinct sub-populations. Although we lack routinely collected population-level risk behavior data and could not adjust for that association in our analysis, we do have data on sexually transmitted diseases, such as rectal gonorrhea, as a proxy for sexual risk behavior. Moreover, a decrease in new cases may reflect testing patterns or risk behavior levels. However, newly diagnosed HIV cases decreased concurrently with a significant increase in HIV testing uptake and a reduction in the number of people unaware of their HIV diagnosis, despite increased cases of rectal gonorrhea. That new HIV infections declined during a period when sexual risk behaviors increased, as suggested by increased cases of rectal gonorrhea, lends further credence to our hypothesis that the reduction in HIV infections was driven primarily by reductions in community viral load rather than reductions in sexual risk behavior. Despite these limitations, we believe our data from multiple independent sources provide persuasive evidence that the recent increases in HIV testing, treatment coverage and efficacy, and viral load suppression are having an effect on the HIV epidemic, and that CVL is a useful biomarker of these effects. In summary, reductions in San Francisco mean and total CVL following comprehensive public health measures to increase HIV testing and ART uptake were consistent with apparent declines in HIV incidence and strongly associated with declines in newly diagnosed HIV infections.

There is current, heated debate on whether “test and treat” strategies can eradicate the HIV epidemic or reduce HIV incidence at a population level. To be clear, we do not suggest that our data prove that treatment alone can accomplish these aims. Rather, we believe achieving a high level of ART coverage is an integral part of a comprehensive “highly active HIV prevention” [54] approach incorporating behavioral, biomedical, public health [55], [56] and structural interventions to the HIV epidemic that has manifest individual health benefits and, we believe, potential prevention effects. While the relative amounts of incidence reduction achievable by behavior change interventions versus VL suppression may be an area of contention, there should be consensus that both are needed. Given the continued HIV transmission that occurs in all areas of the world regardless of whether uptake of ART is high or not, programs intervening on risky behavior (including both sexual and syringe-sharing) also need to be scaled up and improved [54], [57], [58]. And until all of the estimated 9.7 million persons [59] who need HIV treatment are able to access ART, the urgency to scale up treatment remains as a matter of social justice and human rights, regardless of its prevention effects [60]–[63].

In the meantime, we recommend that public health departments and other organizations consider using CVL as an indicator of the overall success of ART uptake and for HIV prevention efforts mediated through increased HIV testing, early linkage into care, improved engagement and retention in care, and increased virologic suppression. Because CVL is temporally upstream of new HIV infections, it may be particularly useful as an outcome measure for multi-level HIV prevention trials or community-level interventions, including behavioral, “test and treat,” “ART as prevention,” or earlier ART initiation [64] strategies at the population level. Additionally, mapping the spatial distribution of CVL may delineate disparities, new “hotspots” or areas with particularly high HIV incidence, allowing a rapid response to target resources and interventions to populations at greatest risk.
